# A Combined Sensing System for Intrusion Detection Using Anti-Jamming Random Code Signals

**DOI:** 10.3390/s22114307

**Published:** 2022-06-06

**Authors:** Hang Xu, Yingxin Li, Cheng Ma, Li Liu, Bingjie Wang, Jingxia Li

**Affiliations:** 1 Key Laboratory of Advanced Transducers and Intelligent Control System, Ministry of Education and Shanxi Province, Taiyuan University of Technology, Taiyuan 030024, China; liyingxin0803@link.tyut.edu.cn (Y.L.); macheng0854@link.tyut.edu.cn (C.M.); liuli01@tyut.edu.cn (L.L.); wangbingjie@tyut.edu.cn (B.W.); lijingxia@tyut.edu.cn (J.L.); 2 College of Physics and Optoelectronics, Taiyuan University of Technology, Taiyuan 030024, China

**Keywords:** intrusion detection, sensing system, random code signal, leaky coaxial cable (LCX) sensor, radar sensor

## Abstract

In order to prevent illegal intrusion, theft, and destruction, important places require stable and reliable human intrusion detection technology to maintain security. In this paper, a combined sensing system using anti-jamming random code signals is proposed and demonstrated experimentally to detect the human intruder in the protected area. This sensing system combines the leaky coaxial cable (LCX) sensor and the single-transmitter-double-receivers (STDR) radar sensor. They transmit the orthogonal physical random code signals generated by Boolean chaos as the detection signals. The LCX sensor realizes the early intrusion alarm at the protected area boundary by comparing the correlation traces before and after intrusion. Meanwhile, the STDR radar sensor is used to track the intruder’s moving path inside the protected area by correlation ranging and ellipse positioning, as well as recognizing intruder’s activities by time-frequency analysis, feature extraction, and support vector machine. The experimental results demonstrate that this combined sensing system not only realizes the early alarm and path tracking for the intruder with the 13 cm positioning accuracy, but also recognizes the intruder’s eight activities including squatting, picking up, jumping, waving, walking forward, running forward, walking backward, and running backward with 98.75% average accuracy. Benefiting from the natural randomness and auto-correlation of random code signal, the proposed sensing system is also proved to have a large anti-jamming tolerance of 27.6 dB, which can be used in the complex electromagnetic environment.

## 1. Introduction

Intrusion detection technology aims to monitor human intruders entering the protected area to prevent their theft and destruction. It has been widely used in the security protection of important places such as warehouses, museums, banks, airports, and transformer substations.

The common intrusion detection technologies include infrared sensors [1,2], video surveillance systems [3,4], electronic fences [5,6,7], vibration cable transducers [8], optical fiber vibration sensors [9,10,11,12], leaky coaxial cable (LCX) sensors [13,14,15,16,17,18,19,20], and radar sensors [21,22,23,24,25].

An infrared sensor is divided into an active infrared sensor and a passive infrared sensor [1,2]. The former emits the invisible infrared light into the linear protected area, and then the intruder blocks the light path between the transceiver to trigger the alarm. The latter continuously locates and tracks the intruder by monitoring his infrared radiation. However, they are vulnerable to the ambient visibility and temperature, respectively. In addition, some floating debris such as leaves and birds block the light path of active infrared sensor, causing false positives. Large shields such as walls also interfere with the detection effect of the passive infrared sensor.

A video surveillance system [3,4] collects images by a camera and extracts intruders from images by the frame difference method or the background subtraction method, so as to detect and track intruders. It can accurately discriminate between the human intruder and animal intruder, but it is susceptible to the ambient visibility and obstructions such as vegetations.

Electronic fences include the pulsed electronic fence [5] and the tension-type electronic fence [6,7]. The former transmits a high-frequency low-voltage pulse to form a closed loop on the fence. If the intruder climbs or damages the fence, it will cause a short circuit or open circuit to give an intrusion alarm. The latter uses the tension detection device to monitor the tension change of wire rope caused by the intruder climbing so as to trigger the alarm. However, wet weather may cause a short circuit fault in the pulsed electronic fence, leading to a false alarm. Long working hours may also cause tension fatigue in the tension detection device. An animal staying for a long time may also cause internal tension of wire rope changes of the tension-type electronic fence to trigger a false alarm.

Vibration cable transducers [8] are usually installed on roofs, walls, fences, or buried underground and realize the intrusion alarm by detecting the vibration of cable surface caused by the intruder climbing. However, they is easily disturbed by vehicle vibration and animal climbing, resulting in false alarms.

Optical fiber vibration sensors mainly include Michelson interferometers [9], Sagnac interferometers [10], Mach-Zehnder interferometers [11] based on interferometric sensors, and phase-sensitive optical time-domain reflectometers [12] based on backscattering. The vibration change of optical fibers induced by the intruder climbing is transformed into the optical phase change so as to realize the intrusion detection. They have long sensing distance and high sensitivity but can easily be disturbed by ambient vibration, and these devices are relatively complex and expensive.

An LCX sensor utilizes a pair of parallel LCXs laid at the protected area boundary to transmit and receive the detection signal, thus forming an electromagnetic field between two LCXs to monitor the intruder. The previous continuous-wave LCX sensor determines that the intruder is present along the LCXs but is unable to locate the intruder within the LCX length [13]. In recent developments, the single frequency pulse [14,15], linear frequency modulation pulse [16,17], and binary phase shift keying pulse [18] have been used as the detection signals of LCX sensors, respectively. The delay time of the above pulse echoing from the intruder is obtained by pulse compression and synchronous subtraction. The pulsed LCX sensors not only have the function of intrusion alarm, but also give the intrusion range along the LCXs. The complementary orthogonal codes based on Golay codes are also transmitted on each of two LCXs to double the intrusion detection range [19] and enhance the signal to noise ratio [20]. The LCXs are buried shallow underground, which are flexibly laid according to terrain, and the electromagnetic field is not interfered by the ambient temperature, visibility, and vibration. However, it can only monitor the protected area boundary and cannot track the intruder inside the area. Additionally, due to the inherent characteristics of detection signals, the LCX sensors are vulnerable to the electromagnetic interferences in free space.

Radar sensors transmit and receive signals by means of antennas, and thus realize intrusion monitoring inside the protected area. These mainly include pulsed Doppler (PD) radars [21], frequency-modulated continuous-wave (FMCW) radars [22,23,24], and FMCW-PD radars [25]. To obtain the intruder’s range and velocity, the PD radar measures the time of arrival and the Doppler frequency shift of echo pulse, and its detection range and resolution depend on the pulse width and power. The FMCW radar adopts the fast Fourier transform and phase comparison techniques for the target echo to obtain the intruder’s range and azimuth, and its unambiguous detection performance is limited by range sidelobes of FMCW signal. The FMCW-PD radar combines the above two radar modes. The FMCW mode and PD mode are flexibly switched for short-range detection and long-range detection, respectively. However, the detection blind spots are easy to form at the protected area boundary due to the weak reflection signal from the long-range intruder. Moreover, it also faces the defect of weak anti-electromagnetic interference ability.

Table 1 summarizes the performance comparison of the above intrusion detection technologies.

In this paper, we propose and experimentally demonstrate a combined sensing system for intrusion detection using anti-jamming random code signals, which combines the LCX sensor and the single-transmitter-double-receivers (STDR) radar sensor. The former is responsible for early intrusion alarm at the protected area boundary; meanwhile, the latter is used for the path tracking and action recognition of intruders inside the protected area. Moreover, the orthogonal physical random code signals generated by the Boolean chaos are used as detection signals, which can be implemented on a field programmable gate array (FPGA) [26]. Compared with the pseudo-random code widely used as the detection signal of time domain reflectometry [27,28], lidar [29,30], and radar [31,32], the random code signal transmitted by our sensing system is the physical random code based on the Boolean chaos. It has properties of natural randomness, aperiodicity, and unpredictability [33], which can eliminate the ambiguous detection caused by the finite length of pseudo-random code. Moreover, its strong anti-jamming ability is also proved by comparing the intrusion detection results with and without noise interference. Compared with the intrusion detection technologies listed in Table 1, the proposed combined sensing system has the following advantages: (1) It is resistant to the ambient temperature, visibility, humidity, vibration, and shelter and has a strong anti-electromagnetic interference ability, so it can monitor the intruder all day. (2) It has multiple detection functions including the early intrusion alarm at the area boundary as well as path tracking and action recognition of the intruder inside the area, so its protection scope covers the area boundary and interior.

This paper is structured as follows. In Section 2, we depict the materials and methods including the experimental setup, the generation and characteristics of the random code signal, and the intrusion detection algorithm. The experimental results are given and analyzed in Section 3. Finally, some discussions and conclusions are outlined in Section 4 and Section 5, respectively.

## 2. Materials and Methods

### 2.1. Experimental Setup

**Figure 1:**
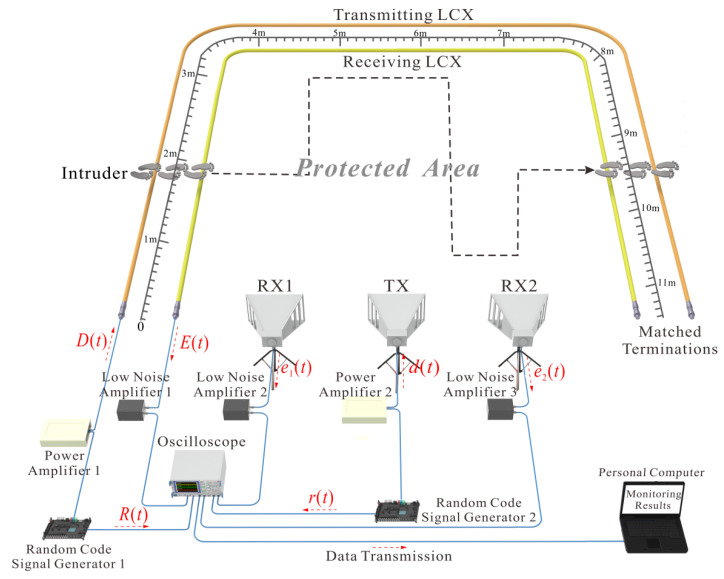
Experimental setup of the combined intrusion-detection sensing system.

**Figure 2:**
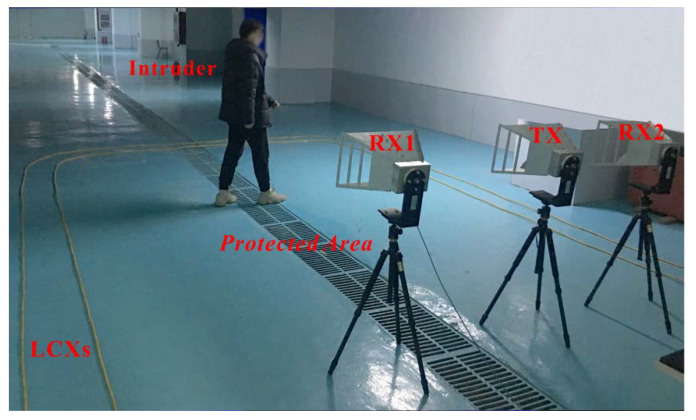
Experimental scene of the intruder entering the protected area.

Figure 1 shows the experimental setup of our combined intrusion-detection sensing system, which integrates the LCX sensor and the STDR radar sensor. In the LCX sensor, the random code signal generator 1 outputs two 500 Mbps random code differential signals. One is amplified by the power amplifier 1 as the detection signal *D*(*t*), while the other serves as the reference signal *R*(*t*). A pair of sparsely braided LCXs laid at the protected area boundary is utilized for transmitting and receiving the random code signal, and thus an electromagnetic field between them is formed to monitor the intruder. The echo signal *E*(*t*), which is composed of the direct waves between two LCXs and the reflected wave from the intruder, is amplified by the low noise amplifier 1, and then collected by an oscilloscope together with *R*(*t*). The 50 Ω matched terminations at the ends of LCXs are used to eliminate end reflections. In the STDR radar sensor, the random code signal generator 2 also outputs two 2 Gbps random code differential signals. One is amplified by the power amplifier 2 as the detection signal *d*(*t*) and transmitted into the protected area by the transmitting antenna (TX), and the other is directly collected by the oscilloscope as the reference signal *r*(*t*). The echo signals *e*_1_(*t*) and *e*_2_(*t*) received by two separate receiving antennas (RXs) 1 and 2 are amplified by two low noise amplifiers 2 and 3, respectively, and then collected by the oscilloscope. The oscilloscope is in the working mode of continuous acquisition and storage for multi-channel signals, which records the intruder’s echo and reference signals at different positions and times. The monitoring data are processed offline and shown on a personal computer. The experimental scene of the intruder entering the protected area is shown in Figure 2. In the experiment, the maximum gain and frequency range of two power amplifiers are 25 dB and 75 Hz–10 GHz, respectively. The maximum gain and frequency range of three low noise amplifiers are 30 dB and 20 MHz–3 GHz, respectively. The TX and RXs are the same broadband horn antennas, and their typical gain, frequency range, and 3 dB beamwidth on E-plane are 11 dBi, 0.5–3 GHz, and 97°–33°, respectively. The bandwidth and characteristic impedance of LCXs are 0.45 GHz and 50 Ω, respectively. The bandwidth and sampling rate of oscilloscope are 2 GHz and 20 GSa/s.

### 2.2. Generation and Characteristics of Random Code Signal

**Figure 3:**
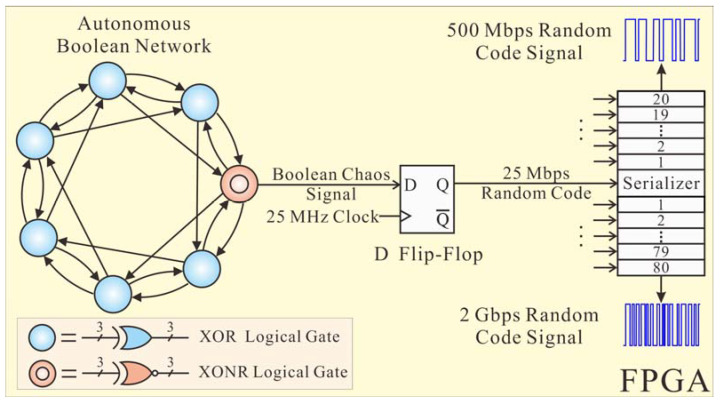
Generation method of the 500 Mbps and 2 Gbps random code signals.

Figure 3 shows the generation method of the random code signal by sampling and time division multiplexing for output signals from chaotic entropy sources, which is realized on an FPGA development board. The chaotic entropy source consists of an autonomous Boolean network with seven nodes, of which six nodes are exclusive-OR (XOR) logical gates and one node is an XNOR (inverse of the XOR) logical gate. Seven nodes form a bidirectional topological ring, and they are connected by adjacent coupling and interval feedback. Firstly, based on the short pulse suppression effect and nonlinear transmission delay characteristics of logical gates in the bidirectional topological ring, the Boolean chaos signal is output from the XNOR logical gate [34]. Secondly, the Boolean chaos signal is sampled through a D flip-flop under the control of a 25 MHz clock, and then the 25 Mbps random code is generated. Finally, by repeating the above structure, the multi-channel 25 Mbps random codes are generated simultaneously and time-division multiplexed via a serializer to improve the code rate of the random code signal. By setting the input channels of a serializer as 20 and 80 channels, the random code signals with code rates of 500 Mbps and 2 Gbps are generated, respectively.

**Figure 4:**
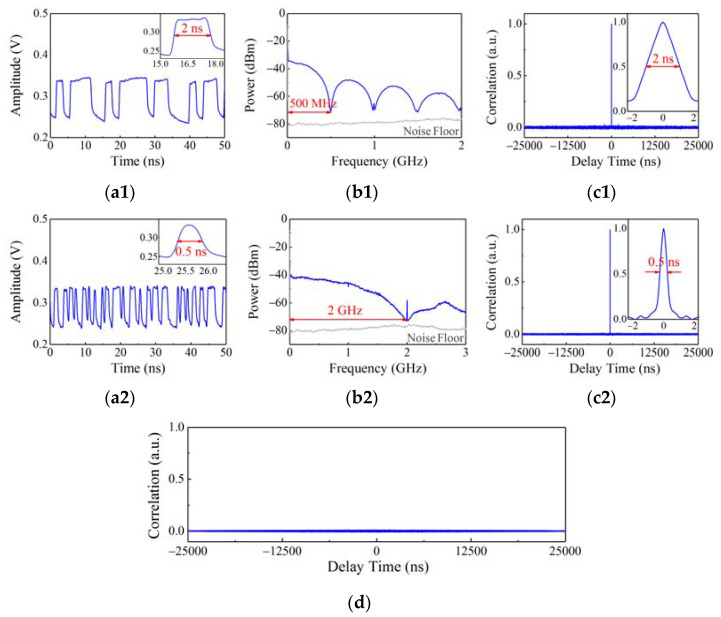
(**a1**,**a2**) Time-domain waveforms; (**b1**,**b2**) power spectrums; (**c1**,**c2**) auto-correlation traces of the 500 Mbps and 2 Gbps random code signals; and (**d**) their cross-correlation trace.

According to the different frequency ranges of TX/RXs and LCXs, the 2 Gbps and 500 Mbps random code signals are selected as the detection signals of STDR radar sensor and LCX sensor, respectively. Figure 4(a1,a2) show their time-domain waveforms, revealing that the high and low levels change rapidly and irregularly. The minimum code widths are 2 ns and 0.5 ns, respectively, as shown in the illustrations. Limited by the 2 GHz oscilloscope bandwidth, the single code shape of 2 Gbps random code signal is close to a sinusoidal pulse. Figure 4(b1,b2) depict the power spectrums of 500 Mbps and 2 Gbps random code signals, respectively. It is shown that their bandwidths are 500 MHz and 2 GHz, respectively, corresponding to their code rates. The random code signals have auto-correlation traces similar to *δ* function, as plotted in Figure 4(c1,c2). A sharp correlation peak and weak sidelobe level appear on their auto-correlation traces as the important basis for correlation ranging. The insets show that the full widths at the half maximums of correlation peaks are 2 ns and 0.5 ns, respectively, which are consistent with their minimum code widths. Figure 4d shows the cross-correlation trace between the 500 Mbps and 2 Gbps random code signals generated by two independent random code signal generators. The cross-correlation coefficient is near zero, indicating that the random code signals generated by the different generators are orthogonal and do not interfere with each other as the detection signals cover the same protected area. Besides, the transmitted power of 500 Mbps and 2 Gbps random code detection signals are 9.8 dBm and 14.8 dBm, respectively.

### 2.3. Intrusion Detection Algorithm

**Figure 5:**
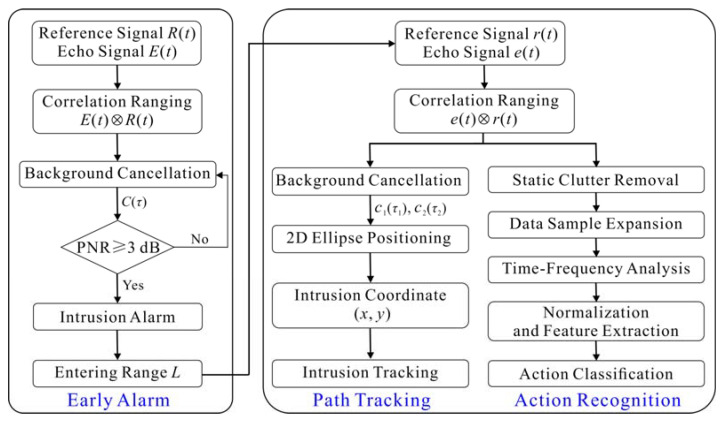
Intrusion detection algorithm of the proposed combined sensing system.

The intrusion detection algorithm used by the proposed combined sensing system is plotted in Figure 5. It firstly uses the LCX sensor to realize the early intrusion alarm at the protected area boundary, and then uses the STDR radar sensor to realize the path tracking and action recognition of the intruder inside the protected area.

#### 2.3.1. Early Alarm

**Figure 6:**
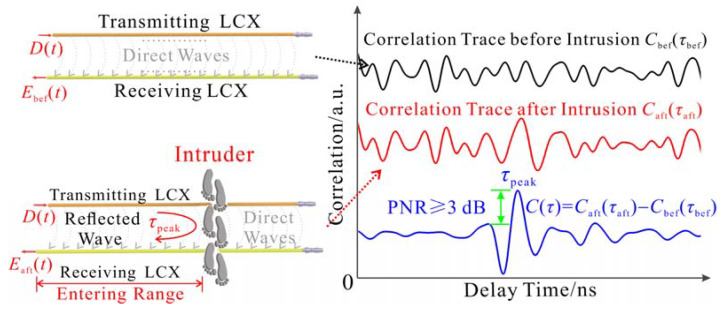
Measurement principle of early alarm.

The outer conductor of LCXs used for early alarm is covered with diamond-shaped holes for transmitting and receiving electromagnetic waves, and an approximate semi-cylindrical monitoring space is formed near the ground surface between two LCXs. The reflected wave from the intruder is received by the adjacent hole together with the direct waves between the LCXs. The correlation traces before and after intrusion are compared to extract the intruder’s range along the LCXs and give an early intrusion alarm. The specific measurement principle is referred to the LCX sensor with chaotic signal in Ref. [35]. As shown in Figure 6, two LCXs are placed parallel to each other for transmitting the detection signal *D*(*t*) and receiving the echo signal *E*(*t*). It is assumed that the echo signals before and after intrusion are *E*_bef_(*t*) and *E*_aft_(*t*), and the corresponding reference signals are *R*_bef_(*t*) and *R*_aft_(*t*). The intrusion detection result *C*(*τ*) from the LCX sensor is obtained by background cancellation that is comparing the correlation traces before and after intrusion *C*_bef_(*τ*_bef_), *C*_aft_(*τ*_aft_), as given by:

(1) $$C_{\mathrm{bef}}\left(\tau_{\mathrm{bef}}\right)\;=E_{\mathrm{bef}}\left(t\right)⊗R_{\mathrm{bef}}\left(t\right)=\underset{T\to \infty }{\lim}\int_{-T/2}^{T/2}E_{\mathrm{bef}}\left(t\right)R_{\mathrm{bef}}^{*}\left(t-\tau_{\mathrm{bef}}\right)dt$$

(2) $$C_{\mathrm{aft}}\left(\tau_{\mathrm{aft}}\right)=E_{\mathrm{aft}}\left(t\right)⊗R_{\mathrm{aft}}\left(t\right)=\underset{T\to \infty }{\lim}\int_{-T/2}^{T/2}E_{\mathrm{aft}}\left(t\right)R_{\mathrm{aft}}^{*}\left(t-\tau_{\mathrm{aft}}\right)dt$$

(3) $$C\left(\tau \right)=C_{\mathrm{aft}}\left(\tau_{\mathrm{aft}}\right)-C_{\mathrm{bef}}\left(\tau_{\mathrm{bef}}\right)\;\;=\delta \left(t-\tau \right)$$

where ⊗ is the cross-correlation calculation, *T* is the integration time, R^*^(*t*) is the complex conjugate of *R*(*t*), and *τ*_bef_ and *τ*_aft_ are the delay time of *E*(*t*) relative to *R*(*t*) before and after intrusion, respectively. Furthermore, the 3 dB peak to noise ratio (PNR) is introduced to automatically determine whether there exists a correlation peak caused by the intruder’s reflected wave in *C*(*τ*), and its expression is:

(4) $$\mathrm{PNR}=10\times \log_{10}\left(\frac{C_{\mathrm{peak}}}{\bar{n}+3\times \mathrm{std}\left(n\right)}\right)$$

where *C*_peak_ is the highest correlation peak in *C*(*τ*) and *n* is the basal sidelobes except *C*_peak_. If PNR < 3 dB, the correlation peak is submerged in the basal sidelobes, indicating that no intrusion occurs. If PNR ≥ 3 dB, the correlation peak is significantly higher than the basal sidelobes, indicating that there exists an intruder entering the protected area by crossing the LCXs. Hence, the early alarm is triggered, and the entering range *L* along the LCXs is shown as:

(5) $$L=v\tau_{\mathrm{peak}}/2$$

where *τ*_peak_ is the roundtrip time between the LCX sensor and intruder along the LCXs, which can be obtained by extracting the delay time of the correlation peak with PNR ≥ 3 dB. *v* is the propagation velocity of the random code signal in the LCXs, which is 0.83*c* (*c* = 3.0 × 10^8^ m/s). A similar situation also occurs when the intruder crosses the LCXs to leave the protected area.

#### 2.3.2. Path Tracking

After the early alarm is issued, the STDR radar sensor adjusts the angle of TX and RXs according to the entering position (*x*_0_, *y*_0_) that is derived by converting *L* into a two-dimensional (2D) coordinate system, so as to ensure that the intruder is within the coverage of the electromagnetic wave. Then, the radar sensor further tracks the intruder’s moving path inside the protected area based on the 2D ellipse positioning principle. In the STDR radar sensor, the detection signal *d*(*t*) is transmitted by the TX, while the RX1 and RX2 are responsible for receiving the echo signals *e*_1_(*t*) and *e*_2_(*t*), respectively. It is assumed that the coordinates of TX, RX1, and RX2 are (*x_p_*, *y_p_*), (*x*_1_, *y*_1_), and (*x*_2_, *y*_2_), respectively. The echo signals from the RX1 and the corresponding reference signals before and after intrusion are *e*_bef1_(*t*), *e*_aft1_(*t*), and *r*_bef1_(*t*), *r*_aft1_(*t*), respectively. The intrusion detection result *c*_1_(*τ*_1_) from the RX1 of STDR radar sensor is obtained by comparing the correlation traces before and after intrusion *c*_bef1_(*τ*_bef1_), *c*_aft1_(*τ*_aft1_), which is expressed as:

(6) $$\begin{array}{ll} c_{1}\left(\tau_{1}\right) & =c_{\mathrm{aft}1}\left(\tau_{\mathrm{aft}1}\right)-c_{\mathrm{bef}1}\left(\tau_{\mathrm{bef}1}\right)\\ & =e_{\mathrm{aft}1}\left(t\right)⊗r_{\mathrm{aft}1}\left(t\right)-e_{\mathrm{bef}1}\left(t\right)⊗r_{\mathrm{bef}1}\left(t\right)\\ & =\delta \left(t-\tau_{1}\right) \end{array}$$

Since *c*_1_(*τ*_1_) is obtained after the early alarm, it is inferred that there exists an obvious correlation peak caused by the intruder’s reflected wave. By extracting the delay time *τ*_peak1_ corresponding to this correlation peak, the range *l*_TX-int-RX1_ between the TX, intruder, and RX1 is deduced as:

(7) $$l_{\mathrm{TX}\text{-}\mathrm{int}\text{-}\mathrm{RX}1}=c\tau_{\mathrm{peak}1}$$

where *τ*_peak1_ represents the delay time of signal propagation between the TX, intruder, and RX1. Similarly, the range *l*_TX-int-RX2_ of TX-intruder-RX2 is also deduced. The intruder’s position coordinate (*x*, *y*) is calculated by jointly solving the following two ellipse equations:

(8) $$\left\{\begin{array}{c} \sqrt{{\left(x-x_{p}\right)}^{2}+{\left(y-y_{p}\right)}^{2}}+\sqrt{{\left(x-x_{1}\right)}^{2}+{\left(y-y_{1}\right)}^{2}}=l_{\mathrm{TX}\text{-}\mathrm{int}\text{-}\mathrm{RX}1}\\ \sqrt{{\left(x-x_{p}\right)}^{2}+{\left(y-y_{p}\right)}^{2}}+\sqrt{{\left(x-x_{2}\right)}^{2}+{\left(y-y_{2}\right)}^{2}}=l_{\mathrm{TX}\text{-}\mathrm{int}\text{-}\mathrm{RX}2} \end{array}\right.$$

Two solutions (*x*, *y*) and (*x*, −*y*) are calculated by the least-squares method [36]. According to the antenna orientation and quadrant of protected area, the false coordinate (*x*, −*y*) is omitted, while the true coordinate is inferred as (*x*, *y*). When the intruder moves inside the protected area, the STRD radar sensor continuously locates and tracks his moving path until he leaves the area, also resulting in an alarm of the LCX sensor.

#### 2.3.3. Action Recognition

When the intruder is in the radial direction of the STDR radar sensor, it is also responsible for recognizing the intruder’s activities to speculate the possible intrusion purpose. The signal processing flow of action recognition is indicated below:

(1) Perform correlation processing on the echo signal *e*_aft2_(*t*) received by the RX2 and the corresponding reference signal *r*_aft2_(*t*) to acquire the correlation trace *c*_aft2_(*τ*_aft2_) after intrusion, as given below:
  (9) $$c_{\mathrm{aft}2}\left(\tau_{\mathrm{aft}2}\right)\;=e_{\mathrm{aft}2}\left(t\right)⊗r_{\mathrm{aft}2}\left(t\right)$$

The delay time axis of *c*_aft2_(*τ*_aft2_) is converted into the range axis by multiplying *c*, and the peak position corresponds to the range between the radar and intruder. The resulting correlation traces are accumulated along the observation time, and then a time-range (TR) matrix **S** is formed.

(2) Remove static clutters caused by the direct waves between the TX and RX2 from **S** by the linear trend subtraction method [37], and then a new TR matrix **Ś** without static clutters is generated.
(3) Extend data sample, i.e., TR matrix **Ś**, to triple itself by time clipping on the observation time, so as to prevent model overfitting and improve system generalization performance.
(4) Perform short-time Fourier transform (STFT) on each range bin of **Ś** to obtain the corresponding time-frequency (TF) matrix **Š***_i_*, and the final TF matrix **Ŝ** is obtained as follows:
  (10) $$Ŝ={Š}_{1}+{Š}_{2}+\ldots +{Š}_{i}\;i=1,\;2,\;\ldots ,n$$
  where *n* is the number of range bins in **Ś**.
(5) Normalize the values of **Ŝ** to between 0 and 1 by Equation (11), so as to eliminate the amplitude sensitivity.
  (11) $${\hat{s}}_{\mathrm{norm}}=\frac{\hat{s}-{\hat{s}}_{\max}}{{\hat{s}}_{\max}-{\hat{s}}_{\min}}$$
  where ŝ∈**Ŝ**, ŝ_norm_ is the normalized value of ŝ, and ŝ_max_ and ŝ_min_ are the maximum and minimum values of ŝ, respectively. On the basis of normalization, the feature extraction for **Ŝ**_norm_ is further implemented by the fast principal component analysis, and its specific process is shown in Ref. [38]. By descending the orders of eigenvalues, the eigenvector of the first eigenvalue, i.e., the first principal component, is selected as the input of action classifier to reduce the data amount and classification complexity without losing the main action information.
(6) Use the support vector machine (SVM) as the intruder’s action classifier, which adopts the LIBSVM with multi-classification function developed by C.-C. Chang and C.-J. Lin [39]. In addition, the radial basis function is selected as the kernel function, and the particle swarm optimization (PSO) is used to find the optimal combination of penalty coefficient *c* and kernel function parameter *g*. Finally, the PSO-SVM model [40] is constructed by adopting the optimal *c* and *g* to recognize the intruder’s activities.

## 3. Experimental Results

### 3.1. Early Alarm and Path Tracking

**Figure 7:**
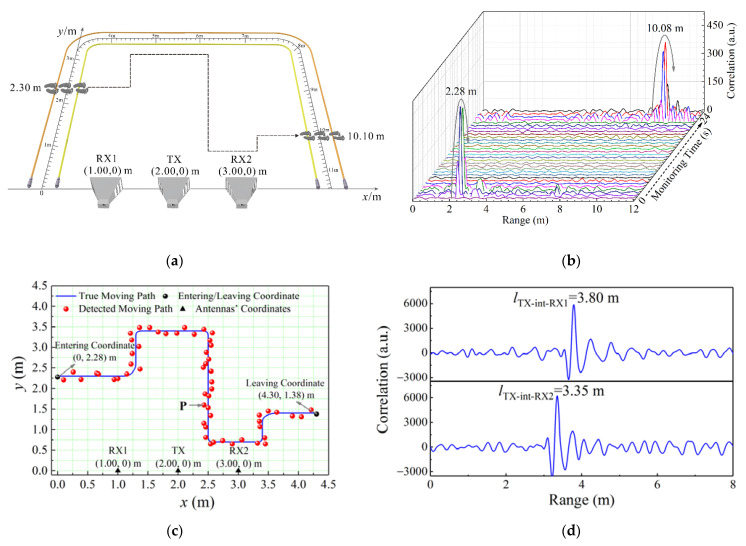
(**a**) Intrusion process, and the detection results of (**b**) early alarm, (**c**) path tracking, and (**d**) ranging at position P.

Figure 7a depicts the intrusion process including the moving path of intruder from entering to leaving the protected area. Two LCXs with the 0.4 m interval are laid at the protected area boundary for transmitting and receiving the 500 Mbps random code signal, and a scaleplate is laid between them to record the true entering and leaving ranges along the LCXs. Besides, the TX, RX1, and RX2 are arranged on the other side of area boundary to transmit and receive the 2 Gbps random code signal. The 2D coordinate system in Figure 7a is established, and the 2D coordinates of TX, RX1, and RX2 are (2.00, 0) m, (1.00, 0) m, and (3.00, 0) m, respectively. An intruder crosses the LCXs at the left boundary to enter the protected area, and the true entering range is given as 2.30 m by the scaleplate. Then, he moves along the path in Figure 7a and finally crosses the LCXs at the right boundary to leave the area. The true leaving range shown by the scaleplate is 10.10 m.

The combined sensing system first uses the LCX sensor to give an early alarm for the area boundary. The detection result of early alarm is shown in Figure 7b. A correlation peak with the 13.8 dB PNR appears at 2.28 m, which is greater than the 3 dB discrimination standard of early alarm, indicating that the intruder enters the protected area at 2.28 m along the LCXs and triggers the early alarm. Compared with the true entering range of 2.30 m, the relative error of ranging is 0.9%. About 24 s later, another correlation peak with a PNR of 12.9 dB arises at 10.08 m, manifesting that the intruder leaves the protected area at 10.08 m with the relative ranging error of 0.2%. The entering and leaving ranges are converted into the 2D coordinate system, and their 2D coordinates are (0.00, 2.28) m and (4.30, 1.38) m, respectively, as shown by the black dots in Figure 7c. It needs to be explained that the closer the intruder gets to the LCXs, the stronger the reflected signal as well as the higher the correlation peak. As the intruder approaches, crosses, and leaves the LCXs, the correlation peak presents the change trend of rising, reaching the highest point, and then falling. Therefore, the range corresponding to the highest correlation peak is taken as the detected entering/leaving range.

After the early alarm is issued, the combined sensing system uses the STDR radar sensor to further track the moving path of intruder. The detected and true moving paths are plotted in Figure 7c. The tracking results show that the intruder enters the protected area from (0.00, 2.28) m, then moves along the red dots inside the area, and finally leaves the area from (4.30, 1.38) m. The ranging results of RX1 and RX2 at position P are shown in Figure 7d. The correlation peaks indicate that the range *l*_TX-int-RX1_ of TX-intruder-RX1 is 3.80 m and the range *l*_TX-int-RX2_ of TX-intruder-RX2 is 3.35 m_._ Two solutions of (2.43, 1.60) and (2.43, −1.60) are obtained by solving Equation (8). According to the antenna orientation and protected area’s quadrant, the intruder’s coordinate is detected as (2.43, 1.60) m. Compared with the true entering coordinate of (2.50, 1.60) m, the absolute positioning error is [(2.50 − 2.43)^2^ + (1.60 − 1.60)^2^]^1/2^ = 7 cm. The detected moving path is consistent with the true moving path of intruder in Figure 7c, and the positioning accuracy of whole path tracking is controlled within 13 cm.

### 3.2. Action Recognition

**Figure 8:**
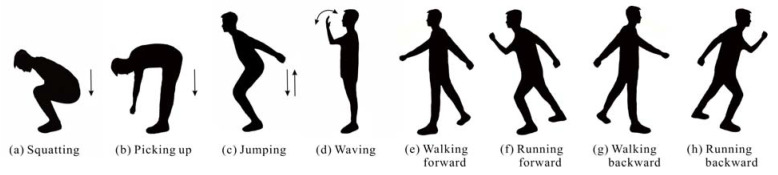
Geometries of the intruder’s eight activities.

The geometries of the intruder’s eight activities are shown in Figure 8, and their echo signals are collected by the RX2. The eight activities include four small range spanning activities such as (a) squatting, (b) picking up, (c) jumping, and (d) waving, and four large range spanning activities such as (e) walking forward, (f) running forward, (g) walking backward, and (h) running backward. As shown in Figure 7c, the data of small range spanning activities are collected at the position P, while the data of large range spanning activities are collected along the straight path through the position P. Eight hundred action samples are collected from five intruders, each of whom repeats each action twenty times. The observation time of all activities is set to 10 s, and it is clipped into 0–8 s, 1–9 s, and 2–10 s, which finally expands the action samples to 2400. The first principal components of each action are disordered randomly and then divided into two parts: 70% as the training set of SVM and 30% as the testing set of SVM.

**Figure 9:**
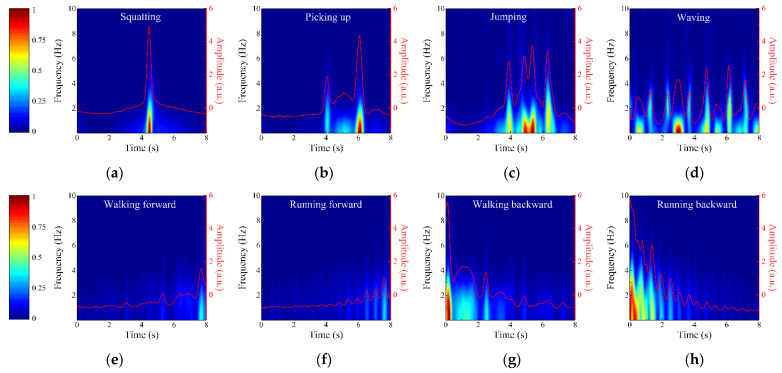
TF diagrams and the corresponding first principal components of (**a**) squatting, (**b**) picking up, (**c**) jumping, (**d**) waving, (**e**) walking forward, (**f**) running forward, (**g**) walking backward, and (**h**) running backward.

Figure 9 shows the TF diagrams of eight activities and the corresponding first principal components represented by red lines. The sudden changes of Doppler frequencies caused by action features are clearly observed from Figure 9. For example, jumping is broken down into four parts including bending knees for power, jumping up, landing, and bending knees for buffer, and their Doppler frequency changes correspond to four peaks at different times in Figure 9c. For large range spanning activities performed in the radial direction of TX and RX2, the closer the intruder is to the antennas, the stronger the echo signal caused by him. Therefore, the activities of walking forward and walking backward can be distinguished by analyzing the change trend in the energy intensity of Doppler frequency with time. Moreover, by comparing Figure 9e,f or Figure 9g,h, it can be found that the occurrence frequency of Doppler frequency peak caused by running is higher than that caused by walking, corresponding to the higher arm swing frequency caused by running. Since the first principal components completely reflect the contour information of TF diagrams and show the feature differences of the actions well, we adopt the first principal components as the input of PSO-SVM classifier to quickly recognize intruder’s activities.

In the PSO-SVM classifier, the optimal scopes of *c* and *g* are 0.1–100 and 0.01–1000, respectively, and the number of particle populations and iterations are 20 and 200, respectively. Then, *c* and *g* are optimized to 5.04 and 0.01 by five-fold cross-validation. The confusion matrix of action recognition is shown in Table 2, indicating that the average recognition accuracy of eight activities reaches 98.75%. Except picking up, jumping, and running forward, the recognition accuracies of other actions reach 100%. The comparison between Figure 9b,c shows that the Doppler frequency features of picking up and jumping are partially similar, so there are a few misjudgments in recognition, but their action recognition accuracies are more than 95%. Finally, the STDR radar sensor is proved to recognize the intruder’s activities.

### 3.3. Anti-Jamming Ability Proof

**Figure 10:**
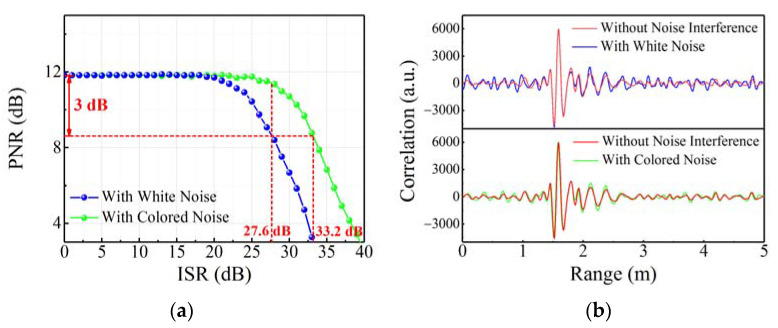
(**a**) Relationship curve between the PNR and ISR; (**b**) comparison results of correlation ranging traces without and with the white/colored noise interference when ISR = 27.6 dB.

In order to demonstrate the anti-jamming ability of the random code signal, the white noise and the colored noise with different amplitudes as the interference signals are mixed into the echo signal of the STDR radar sensor that transmits the 2 Gbps random code signal as the detection signal. On the one hand, the interference-to-signal ratio (ISR) is introduced to characterize the intensity of noise interference, as given by:

(12) $$\mathrm{ISR}=10\times \log_{10}\left(\sigma /\mu \right)$$

where *σ* is the standard deviation of noise interference, and *µ* is the mean value of random code echo signal in the absence of noise interference. On the other hand, the PNR is also used to quantify the influence of noise interference on the correlation ranging of random code signal. The relationship curves between the PNR and ISR under two kinds of noise interference are depicted in Figure 10a, indicating that the PNRs of both decrease slowly with the increase in ISR. The interference level between signals can be qualitatively analyzed by the overlapping scope of frequency band on their power spectrums, and the larger overlapping scope corresponds to more serious interference. Compared with the narrow-band colored noise, the white noise obviously has the larger overlapping scope of frequency band with the random code signal. Therefore, its interference to the random code signal is more serious, resulting in the faster PNR decline, which is the focus of the following research. When the PNR decreases by 3 dB from the maximum value, the ISR under the white noise interference, as the anti-jamming tolerance, reaches 27.6 dB. Figure 10b shows the comparison results of correlation ranging traces without and with the white/colored noise interference under the 27.6 dB ISR. The results show that even if the ISR reaches 27.6 dB, the noise interference cannot affect the correlation peak at 1.60 m representing the intruder’s range, and the basal sidelobes caused by the white noise are slightly higher than those caused by the colored noise. Therefore, the results of early alarm and path tracking based on the correlation ranging of the random code signal will not be disturbed.

**Figure 11:**
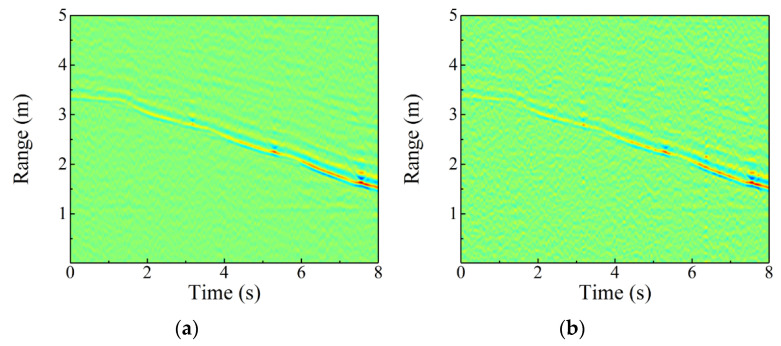
TR diagrams of walking forward (**a**) without and (**b**) with the white noise interference when ISR = 27.6 dB.

**Figure 12:**
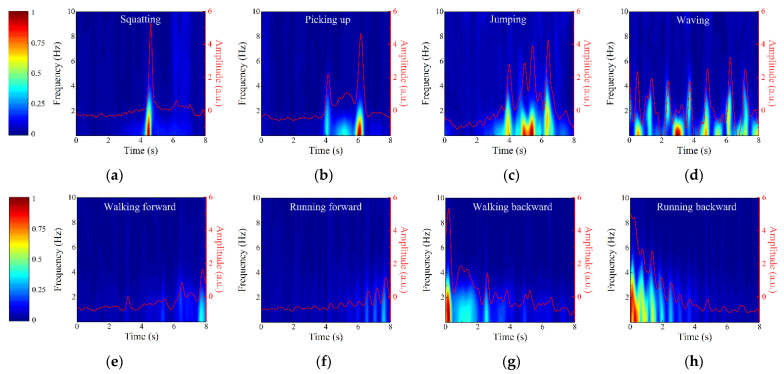
TF diagrams and the corresponding first principal components of (**a**) squatting, (**b**) picking up, (**c**) jumping, (**d**) waving, (**e**) walking forward, (**f**) running forward, (**g**) walking backward, and (**h**) running backward when ISR = 27.6 dB.

Figure 11 also gives the TR diagrams of walking forward without and with the white noise interference under the 27.6 dB ISR; it is concluded that the TR diagram is not affected by the interference. Therefore, the TF diagram obtained by STFT of TR diagram is also undisturbed. Figure 12 shows the TF diagrams and first principal components of eight activities when the ISR is 27.6 dB. Compared with Figure 9, although the white noise interference introduces a small amount of background clutter, the main characteristics of eight activities are still complete, which lays a foundation for ensuring the accuracy of action recognition.

Furthermore, the correlation coefficient (CC) is utilized to quantify the influence of white noise interference on the TF diagram. It is assumed that the data matrices of TF diagrams with and without the white noise interference are **M** and **N**, respectively, where $\hat{S}={\left\{\hat{s}\left(x_{i},y_{j}\right)\right\}}_{i=1,j=1}^{m,n}$, **Ŝ** = **M**, **N**, *a* = *m*, *n*, *a*(*x_i_*, *y_j_*) represents the gray value of a point on the two diagrams. Then, the CC between **M** and **N** is calculated as follows:

(13) $$\mathrm{CC}=\frac{\sum_{i=1}^{m}\sum_{j=1}^{n}m\left(x_{i},y_{j}\right)n\left(x_{i},y_{j}\right)}{{\left[\sum_{i=1}^{m}\sum_{j=1}^{n}m^{2}\left(x_{i},y_{j}\right)\times \sum_{i=1}^{m}\sum_{j=1}^{n}n^{2}\left(x_{i},y_{j}\right)\right]}^{1/2}}$$

**Figure 13:**
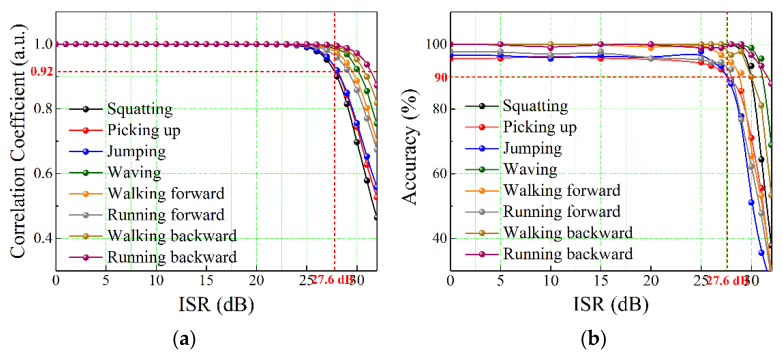
Variation curves of (**a**) CC and (**b**) recognition accuracy for eight activities with the increase in ISR.

Since the CC is a statistical correlation, the closer the CC is to 1, the more similar the two diagrams are, and the less influence the white noise interference has on the TF diagram. Figure 13a shows the variation curves of CC with the ISR increase for eight activities. The results show that the CC values of eight activities initially remain unchanged with the increase in ISR, and then decrease slowly when the ISR exceeds 25 dB, and finally remain at 0.92 even if the ISR increases to 27.6 dB. Furthermore, Figure 13b shows the variation curves of recognition accuracies for eight activities with the increase in ISR. Compared with Figure 13a, the change rule of recognition accuracy is consistent with the variation trend of CC. Although the recognition accuracies decrease slowly when the ISR exceeds 25 dB, the recognition accuracies of all activities remain 90% under the 27.6 dB ISR. When the ISR continues to increase, the recognition accuracies drop to below 90%, indicating that the STDR radar sensor cannot accurately identify the action type of intruder. The combined sensing system is finally proved to realize the anti-jamming early intrusion alarm, path tracking, and action recognition benefiting from the correlation ranging of the random code signal.

## 4. Discussion

The proposed intrusion-detection sensing system not only combines the functions of LCX sensor and radar sensor, but also has been proven to be more resistant to the external electromagnetic interference benefitting from the anti-jamming of random code signal. In addition, it theoretically has a higher range resolution than the LCX sensor and radar sensor reported in some of the literature. The range resolution is obtained by *v*/2*B*, where *v* is the propagation velocity of detection signal in different media, *B* is the effective bandwidth of detection signal. In our sensing system, *v* = 0.83*c* in LCXs for the LCX sensor, while *v* = *c* in free space for the STDR radar sensor. Limited by the 0.45 GHz bandwidth of LCXs, the effective bandwidth of 500 Mbps random code signal is 0.45 GHz. Thus, the LCX sensor can achieve the 28 cm range resolution that is better than the 6.64 m range resolution in Ref. [16] and 3.32 m range resolution in Ref. [17]. In addition, since the frequency range of TX and RXs are 0.5–3 GHz, the effective frequency band of 2 Gbps random code signal is 0.5–2 GHz with the 1.5 GHz effective bandwidth. Therefore, the STDR radar sensor can realize the range resolution of 10 cm, which is better than the 30 cm range resolution in Ref. [24].

## 5. Conclusions

This paper develops a combined intrusion-detection sensing system, which combines the LCX sensor and the STDR radar sensor and takes the random code signals as the detection signals. Compared with the existing LCX sensors and radar sensors for intrusion detection, its advantages are as follows: (1) Early alarm at the area boundary, as well as path tracking and action recognition inside the area, which are implemented simultaneously. (2) Based on the broadband characteristic of random code signal, the positioning accuracy of 13 cm and the action recognition accuracy of 98.75% are realized. (3) Benefitting from the correlation ranging of the random code signal, the anti-jamming ranging, path tracking, and action recognition are realized, and the anti-jamming tolerance reaches 27.6 dB.

The combined intrusion-detection sensing system is proved to be feasible for early warning, path tracking, and action recognition of a single intruder in the ideal indoor environment. In future work, we aim to realize the simultaneous detection of multiple intruders in the complex outdoor environment. The cross-path matching of multiple intruders, the action discrimination of multiple intruders at the same time, and the interference of complex environmental factors will be the focus of follow-up research. It is reasonably believed that this research provides a promising solution for area safety protection in the complex electromagnetic environment.

## Figures and Tables

**Table 1:** Performance comparison of the existing intrusion detection technologies.

Intrusion Detection Technologies		Intruder Location	Multiple Intrusion Detection	Intruder Tracking Inside the Area	Ambient Interference
Infrared Sensor	Active	No	No	No	Visibility and Floating Debris
	Passive	Yes	Yes	Yes	Temperature and Large Shields
Video Surveillance System		Yes	Yes	Yes	Visibility and Obstructions
Electronic Fence	Pulsed	Yes	No	No	Humidity in Wet Weather
	Tension	No			Animal Climbing
Vibration Cable Transducer		No	No	No	Vehicle Vibration and Animal Climbing
Optical Fiber Vibration Sensor		Yes	Yes	No	Ambient Vibration
LCX Sensor		Yes	Yes	No	Electromagnetic Waves
Radar Sensor		Yes	Yes	Yes	Electromagnetic Waves

**Table 2:** The confusion matrix of action recognition.

Pred/True (%)	(a)	(b)	(c)	(d)	(e)	(f)	(g)	(h)
(a)	100	0	0	0	0	0	0	0
(b)	0	95.56	4.44	0	0	0	0	0
(c)	1.11	2.22	96.67	0	0	0	0	0
(d)	0	0	0	100	0	0	0	0
(e)	0	0	0	0	100	0	0	0
(f)	0	0	0	0	2.22	97.78	0	0
(g)	0	0	0	0	0	0	100	0
(h)	0	0	0	0	0	0	0	100

## Data Availability

The data presented in this paper will be made available on request via the corresponding author’s email with appropriate justification.

## References

[1] Aldalahmeh S.A., Hamdan A.M., Ghogho M., McLernon D. Enhanced-Range Intrusion Detection Using Pyroelectric Infrared Sensors. Proceedings of the Sensor Signal Processing for Defence (SSPD).

[2] Sahoo K.C., Pati U.C. IoT based intrusion detection system using PIR sensor. Proceedings of the 2nd IEEE International Conference on Recent Trends in Electronics, Information & Communication Technology (RTEICT).

[3] Wang J.-X. Research and implementation of intrusion detection algorithm in video surveillance. Proceedings of the International Conference on Audio, Language and Image Processing (ICALIP).

[4] Chen H., Chen D.F., Wang X.F. Intrusion detection of specific area based on video. Proceedings of the 9th International Congress on Image and Signal Processing, BioMedical Engineering and Informatics (CISP-BMEI).

[5] Yang S., Bu F.L. Design of Frequency Adjustable Pulse in Intelligent Electronic Fence System. Proceedings of the 2nd International Conference on Telecommunications and Communication Engineering.

[6] Tang Z.J., Liu P.L., Luo Q., Ye Z.F. (2014). Main Control System Design for Balanced Tension-Type Electronic Fence. Appl. Mech. Mater..

[7] Tang Z.J., Li T.L., Leng X.N. (2015). The hybrid time-frequency detection method based on EEMD for balanced tension-type electronic fence. Appl. Technol. Innov..

[8] Sun X.R., Ma H.L., Du X.H. (2011). Round-Guard System Based on the Vibration Cable Transducer. Adv. Mater. Res..

[9] Hong X.B., Wu J., Zuo C., Liu F.S., Guo H.X., Xu K. (2011). Dual Michelson interferometers for distributed vibration detection. Appl. Opt..

[10] Pi S.H., Wang B.J., Jia B., Sun Q., Xiao Q., Zhao D. (2015). Intrusion localization algorithm based on linear spectrum in distributed Sagnac optical fiber sensing system. Opt. Eng..

[11] Liu K., Tian M., Jiang J.F., An J.C., Xu T.H., Ma C.Y., Pan L., Wang T., Li Z.C., Zheng W.J. (2016). An Improved Positioning Algorithm in a Long-Range Asymmetric Perimeter Security System. J. Lightw. Technol..

[12] Zhan Y.G., Song Z.K., Sun Z.Y., Yu M.H., Guo A.J., Feng C.H., Zhong J.X. (2021). A distributed optical fiber sensor system for intrusion detection and location based on the phase-sensitive OTDR with remote pump EDFA. Optik.

[13] Harman R.K. Intrepid MicroTrack leaky cable sensor. Proceedings of the 36th Annual IEEE International Carnahan Conference on Security Technology.

[14] Guan Q., Lu H.M., Chen C.C., Wang K.B. (2019). Research on human intruder detection and localization based on LCX sensor. Int. J. RF Microw. Comput. Aided Eng..

[15] Guan Q., Lu H.M., Wang K.B., Chen C.C. (2018). A Novel Approach for Intruder Localization Based on Leaky Coaxial Cable Sensor with IQ Demodulation and Synchronous Subtraction. Appl. Comput. Electromagn. Soc. J..

[16] Guan Q., Chen C.C., He C.X. (2018). A novel sensor using VHF zigzag-slotted leaky coaxial cable for intruder localization. Microw. Opt. Technol. Lett..

[17] Guan Q., Lu H.M., Chen C.C., He C.X. (2018). Multiple Human Targets Detection and Localization Using Leaky Coaxial Cable Sensing Technique. Appl. Comput. Electromagn. Soc. J..

[18] Liu Y., Guan Q., Lu H.M., Tan K.B., Lan Y.R. (2018). A New Location Method of Leaky Coaxial Cable Sensor Based on Pulse Compression. J. Microw..

[19] Harman K., Hodgins B. The next generation of GUIDAR technology. Proceedings of the 38th Annual IEEE International Carnahan Conference on Security Technology.

[20] Harman K., Hodgins B., Patchell J. Experience with Ranging Buried Cable Sensing. Proceedings of the 41st Annual IEEE International Carnahan Conference on Security Technology.

[21] Foley E., Harman K., Cheal J. (2002). Improving intrusion detection radar. IEEE Aerosp. Electron. Syst. Mag..

[22] Butler W., Poitevin P., Bjomholt J. Benefits of Wide Area Intrusion Detection Systems using FMCW Radar. Proceedings of the 41st Annual IEEE International Carnahan Conference on Security Technology.

[23] Martínez F.P., Galeano F.C. New microwave sensors for intrusion detection systems. Proceedings of the IEEE 33rd Annual International Carnahan Conference on Security Technology (Cat. No.99CH36303).

[24] Xiong D.D., Cui G.L., Feng L.F., Yi W., Kong L.J., Yang X.B. A location and tracking method for indoor and outdoor target via multi-channel phase comparison. Proceedings of the 20th International Conference on Information Fusion (Fusion).

[25] Im Y.-T., Park S.-O. (2013). An FMCW and chirp pulse-doppler radar system for surveillance in X-band. Microw. Opt. Technol. Lett..

[26] Rosin D.P., Rontani D., Gauthier D.J. (2013). Ultrafast physical generation of random numbers using hybrid Boolean networks. Phys. Rev. E.

[27] Okada K., Hashimoto K., Shibata T., Nagaki Y. (1980). Optical cable fault location using correlation technique. Electron. Lett..

[28] Sharma C.R., Furse C., Harrison R.R. (2007). Low-Power STDR CMOS Sensor for Locating Faults in Aging Aircraft Wiring. IEEE Sensors J..

[29] Zhang Y.F., He Y., Yang F., Luo Y., Chen W.B. (2016). Three-dimensional imaging lidar system based on high speed pseudorandom modulation and photon counting. Chin. Opt. Lett..

[30] She C.-Y., Abo M., Yue J., Williams B.P., Nagasawa C., Nakamura T. (2011). Mesopause-region temperature and wind measurements with pseudorandom modulation continuous-wave (PMCW) lidar at 589 nm. Appl. Opt..

[31] Aftanas M., Zaikov E., Drutarovský M., Sachs J. Through Wall Imaging of the Objects Scanned by M-sequence UWB Radar System. Proceedings of the 18th International Conference Radioelektronika.

[32] Xia Z.H., Fang G.Y., Ye S.B., Zhang Q.Y., Chen C., Yin H.J. (2014). A novel handheld pseudo random coded UWB radar for human sensing applications. IEICE Electron. Exp..

[33] Gong L.S., Zhang J.G., Sang L.X., Liu H.F., Wang Y.C. (2020). The Unpredictability Analysis of Boolean Chaos. IEEE Trans. Circuits Syst. II Exp. Briefs.

[34] Zhang R., Cavalcante H.L.D.D.S., Gao Z., Gauthier D.J., Socolar J.E.S., Adams M.M., Lathrop D.P. (2009). Boolean chaos. Phys. Rev. E.

[35] Xu H., Qiao J., Zhang J.G., Han H., Li J.X., Liu L., Wang B.J. (2018). A High-Resolution Leaky Coaxial Cable Sensor Using a Wideband Chaotic Signal. Sensors.

[36] Svecová M., Kocur D., Zetik R. Object Localization Using Round Trip Propagation Time Measurements. Proceedings of the 18th International Conference Radioelektronika.

[37] Xu H., Li L.Q., Li Y., Zhang J.G., Han H., Liu L., Li J.X. (2019). Chaos-Based Through-Wall Life-Detection Radar. Int. J. Bifurc. Chaos.

[38] Li X.-Y., Lin Z.-X. Face Recognition Based on HOG and Fast PCA Algorithm. Proceedings of the Fourth Euro-China Conference on Intelligent Data Analysis and Applications.

[39] Chang C.-C., Lin C.-J. (2011). LIBSVM: A library for support vector machines. ACM Trans. Intell. Syst. Technol..

[40] Long Y., Du Z.-J., Wang W.-D., Zhao G.-Y., Xu G.-Q., He L., Mao X.-W., Dong W. (2016). PSO-SVM-Based Online Locomotion Mode Identification for Rehabilitation Robotic Exoskeletons. Sensors.

